# Of Revolutions and Roadblocks: The Emerging Role of Machine Learning in Biocatalysis

**DOI:** 10.1021/acscentsci.5c00949

**Published:** 2025-09-15

**Authors:** Tobias Vornholt, Peter Stockinger, Mojmír Mutný, Markus Jeschek, Bettina Nestl, Gustav Oberdorfer, Silvia Osuna, Jürgen Pleiss, Ditte Hededam Welner, Andreas Krause, Rebecca Buller, Thomas R. Ward

**Affiliations:** † Department of Chemistry, 27209 University of Basel, 4058 Basel, Switzerland; ‡ National Centre of Competence in Research (NCCR) Molecular Systems Engineering, 4056 Basel, Switzerland; § Competence Center for Biocatalysis, Zurich University of Applied Sciences, Einsiedlerstrasse 31, Wädenswil 8820, Switzerland; ∥ National Centre of Competence in Research (NCCR) Catalysis, 8092 Zurich, Switzerland; ⊥ Eric and Wendy Schmidt Center, Broad Institute, 75 Ames Street, Cambridge, Massachusetts 02142, United States; # Department of Computer Science, ETH Zurich, 8092 Zurich, Switzerland; 7 Laboratory of Synthetic and Applied Microbiology (LSAM), SB ISIC & SV IBI, École Polytechnique Fédérale de Lausanne (EPFL), 1015 Lausanne, Switzerland; 8 Innophore GmbH, Am Eisernen Tor 3, 8010 Graz, Austria; 9 Institute of Biochemistry, Graz University of Technology, Petersgasse 12/2, 8010 Graz, Austria; 10 Institut de Química Computacional i Catàlisi and Departament de Química, Universitat de Girona, 17003 Girona, Spain; 11 ICREA, 08010 Barcelona, Spain; 12 Institute of Biochemistry, 9149 University of Stuttgart, Allmandring 31, 70569 Stuttgart, Germany; 13 DTU Biosustain, 5205 Technical University of Denmark, Lyngby 2800, Denmark; 14 Department of Chemistry, Biochemistry and Pharmaceutical Sciences, University of Bern, 3012 Bern, Switzerland

## Abstract

Machine learning (ML) is rapidly turning into a key technology for biocatalysis. By learning patterns in amino acid sequences, protein structures, and functional data, ML models can help navigate complex fitness landscapes, uncover new enzymes in databases, and even design biocatalysts *de novo*. Along with advances in DNA synthesis and sequencing, laboratory automation, and high-throughput screening, ML is increasing the speed and efficiency of enzyme development. In this Outlook, we highlight recent applications of ML in the fields of enzyme discovery, design, and engineering, with a focus on current challenges and emerging solutions. Furthermore, we discuss barriers that impede a broader and faster adoption of ML-based workflows in the biocatalysis community. We conclude by suggesting best practices for fostering effective collaborations in this interdisciplinary field.

## Graphical abstract

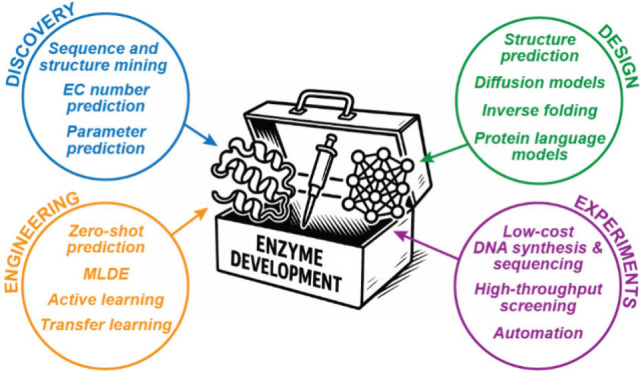

## Introduction

In just a few years, the landscape of protein design and engineering has experienced a remarkable transformation. Among many breakthroughs, we have witnessed the development of tailor-made protein binders capable of neutralizing snake venom,^1^ protein nanoparticle vaccines that protect against emerging viruses,^2^ and synthetic sensors for the detection of small molecules.^3^ These developments are the result of synergistic advances across several technological frontiers. In particular, the integration of machine learning (ML) into tasks like protein structure prediction, *de novo* design, and sequence-function mapping has significantly enhanced the capabilities of computational tools in protein science. In parallel, the decreasing cost of DNA synthesis and sequencing, as well as advances in high-throughput screening and lab automation, greatly facilitate the characterization of large numbers of proteins, which in turn yields valuable training data for ML models.

These developments also have a profound impact on biocatalysis. Enzyme development has long relied on the optimization of natural enzymes by directed evolution, which has enabled numerous industrial applications of biocatalysis.^4,5^ Over time, this workflow has increasingly been augmented by computational tools. This transformation has accelerated with the introduction of ML techniques, which hold considerable promise in addressing some of the most significant challenges within biocatalysis.^6^ Key tasks in this field include the identification of promising enzymes in databases (enzyme discovery), the *de novo* design of entirely new enzymes (enzyme design), and the optimization of existing enzymes with respect to important properties (enzyme engineering). ML tools already often outperform previous approaches in enzyme design,^7^ and numerous studies have demonstrated the utility of ML for tasks such as enzyme discovery or engineering^8^ (see Figure 1 for an overview of common ML-assisted workflows). At the same time, compared to the development of proteins for tasks like binding, enzymes present additional challenges for both computational and experimental approaches, for example due to the complexity of enzymatic mechanisms and the need for specialized analytical techniques. Thus, in many cases bespoke solutions are required to further enhance and accelerate enzyme development and render biocatalysis more widely applicable. Here, we provide an overview of new paradigms in enzyme development, with a particular emphasis on current challenges and emerging solutions. We consider computational tools, experimental technologies, as well as the sociocultural factors influencing the future trajectory of this field. For a more detailed discussion of specific topics, we refer the reader to recent reviews on enzyme discovery,^9^ enzyme design,^7,10^ and computational enzyme engineering.^8,11,12^

**Figure 1:**
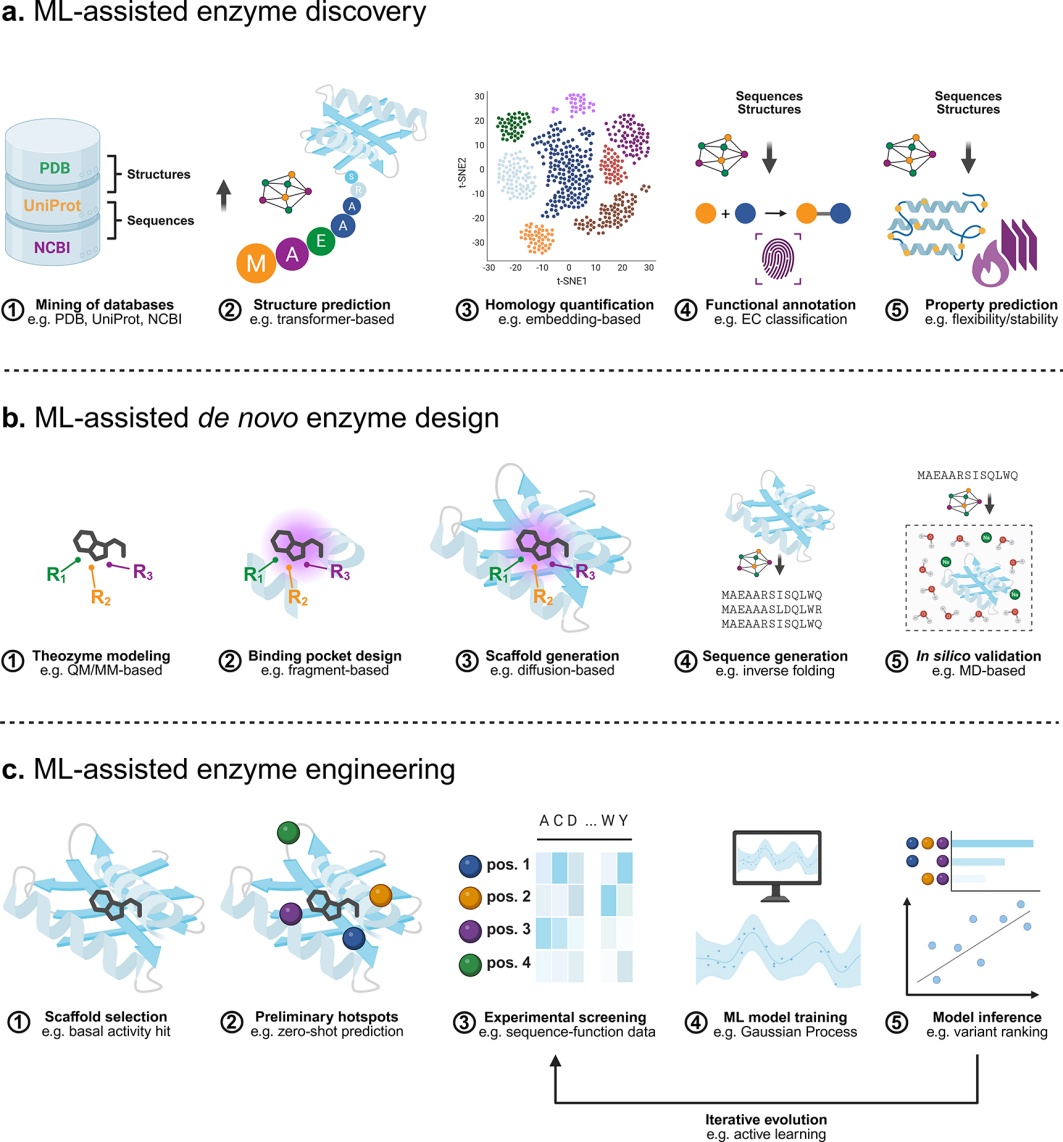
Exemplary ML-assisted workflows for the discovery, *de novo* design, and engineering of enzymes. (a) Enzyme discovery often begins with the mining of databases for enzymes that share some similarity to a template. This is aided by the facile structure prediction enabled by ML tools. Homology can be quantified using various metrics, including ML-based embeddings. Candidate sequences can also be evaluated based on predicted EC numbers and predicted properties such as thermal stability or ease of recombinant expression. (b) ML-assisted *de novo* enzyme design typically requires a model of a minimal active site, around which a protein backbone is designed. Subsequently, a compatible amino acid sequence is designed using an inverse folding model. Various *in silico* tools can then be employed to validate designs and select variants for experimental testing. (c) ML-assisted enzyme engineering is used to improve the activity (or other properties) of a given enzyme. This can involve the use of zero-shot ML methods to identify hotspots or promising mutations without the need for experimental data. Subsequently, selected variants are tested experimentally. The resulting sequence-function data are used to train a model, which can be used to predict the properties of unseen variants. Frequently, several rounds of experimental testing and modeling are performed.

## Emerging Paradigms in Enzyme Development

### Enzyme Discovery

Modern genome and metagenome projects have generated extensive repositories of protein sequences, numbering in the hundreds of millions.^13^ However, only a small fraction of these proteins has been experimentally characterized. Clearly, these sequence databases contain a multitude of enzymes with useful properties, but linking the sequences to their corresponding functions is challenging. Current initiatives aimed at addressing this issue are pursuing different approaches. On the one hand, automatically annotating sequences with functional attributes has been a longstanding goal in the field. On the other hand, researchers interested in a specific reaction can mine databases for enzymes that are likely to catalyze this reaction. Historically, both endeavors have relied primarily on sequence or structural similarity to known enzymes.

Enzyme mining typically requires a query sequence that has been shown to catalyze the reaction of interest. This sequence can be used to search for related sequences using BLAST,^14^ or more recent and faster methods such as MMseqs2.^15^ This step will typically yield hundreds or even thousands of sequence hits, which need to be filtered based on expert domain knowledge about active sites or other determinants of the desired property. This process can be partially automated using tools like EnzymeMiner,^16^ which combines sequence-based homology searches with filtering based on user-specified essential residues. To assist users in selecting variants for experimental testing, several additional properties are predicted, such as the likelihood of expressing the enzyme in soluble form in *Escherichia coli*.

Until recently, the search for enzymes based on structural motifs was restricted to a comparatively small number of proteins with experimentally determined structures. However, breakthroughs in protein structure prediction using ML, along with databases such as AlphaFold DB^17^ or ESM Metagenomic Atlas^18^ and structural homology search tools such as FoldSeek,^19^ now enable structure-based mining at unprecedented scale. This development is very promising, as structure is more conserved than sequence during evolution.^20^ The search can also focus on active site properties, facilitating the discovery of promiscuous enzymes that share similar active sites but very different sequences and folds.^21^ Despite these advances, the need for a template enzyme with rudimentary activity for the desired reaction (or a closely related one) remains a limitation of current enzyme mining approaches.

High-quality, standardized data sets remain a bottleneck for generalizable ML models of enzymatic properties.

Parallel efforts to reliably annotate enzyme sequences in databases can benefit enzyme mining projects and provide insights into various questions in basic research. Over the past years, numerous ML models have been developed that predict Enzyme Commission (EC) numbers from sequence. A particularly noteworthy example is a model dubbed CLEAN,^22^ which uses a contrastive learning framework to embed enzyme sequences in a space where distance correlates with functional similarity. This method, combined with powerful protein language model embeddings, has achieved a high accuracy in EC number prediction and was able to predict promiscuous activity. Other models also take predicted structures into consideration. For example, GraphEC^23^ uses geometric graph learning and first predicts the location of the active site, followed by EC number prediction. This led to further gains in accuracy.

Besides EC numbers, models have been developed to predict other relevant properties such as kinetic parameters.^24,25^ As generalist models may not always achieve the required level of detail or accuracy, task-specific models have been developed to predict the properties of enzymes from specific families (e.g., glycosyltransferases^26,27^ ). However, such approaches remain challenging due to the limited availability of functional data, the multifunctionality of enzymes,^28^ as well as inconsistent experimental procedures and reporting practices. Recent advances in automated reaction profiling promise to enhance the robustness of functional annotations, particularly in the context of revealing enzyme promiscuity.^29^

In general, data-driven modeling paradigms rely on the accessibility, quantity, and quality of experimental data for training. The success of structure prediction by ML models is largely attributable to the availability of large, structured, consistent, and high-quality data sets on protein sequences and structures. In contrast, quantitative functional data—such as *k*_cat_ or *K*_M_ values of enzyme-catalyzed reactions—are available only for a relatively small number of enzymes. Moreover, the fraction of enzyme sequences with functional experimental characterization is biased toward certain enzyme classes (e.g., hydrolases), while others are underrepresented. Another limitation is the accuracy of experimental annotations – enzyme databases often contain errors or lack critical metadata and details on data processing, which results in published values that may not be reproducible. Thus, it would be important to adopt standardized reporting practices (such as the STRENDA guidelines^30,31^ ) and data formats (such as the markup language EnzymeML^32^). Similarly, the reporting of model performance can be inconsistent, but community-driven standards are currently emerging.^33^

### Enzyme Design

Early examples of *de novo* enzyme design typically centered on conceptualizing an idealized active site – referred to as a theozyme – consisting of key catalytic residues positioned around a reaction’s transition state. This was followed by a search for native protein scaffolds capable of accommodating this active site using tools from the Rosetta macromolecular modeling suite, in some cases followed by short molecular dynamics (MD) simulations. This paradigm saw success in designing enzymes for reactions such as Kemp elimination,^34^ retro aldol reactions,^35^ and Diels–Alder reactions^36^ (among others), however, the activity of the resulting enzymes was low. Accordingly, extensive directed evolution efforts were required to turn the designed enzymes into proficient biocatalysts.^37,38^ These early *de novo* designs were restricted by the limited number of naturally occurring, characterized scaffolds as well as the insufficient accuracy of design tools.

The significant leap in the accuracy of protein structure predictions enabled by deep learning tools such as AlphaFold2 ^39^ and RosettaFold^40^ has profoundly influenced the field of enzyme design. On the one hand, these computational tools can be used to validate designs *in silico*, enabling researchers to focus experimental efforts on candidates most likely to adopt the desired fold. On the other hand, structure prediction models can be used to “hallucinate” novel structures by iteratively optimizing a random input sequence until it is predicted to fold into a stable protein.^41^ This process can be constrained in such a way that it generates a *de novo* scaffold around a desired motif, such as an enzyme active site.^42^ For example, constrained hallucination has been combined with Rosetta-based design to create novel luciferases exhibiting high activity and selectivity, although experimental screening of several thousand variants was still required.^43^

More recently, RosettaFold has been refined for protein structure denoising tasks to create a generative model dubbed RFdiffusion.^44^ By starting from random noise, this model can generate plausible backbone structures that can be conditioned on various constraints, including binding sites or specific motifs. Compared to hallucination, RFdiffusion is more computationally efficient and offers ways of biased sampling to provide specific target structures. Further improvements were introduced with RFdiffusion2,^45^ which can generate enzymes directly from atom-level active site descriptions and was able to design scaffolds around a greater variety of active sites than RFdiffusion (41/41 vs 16/41) on an *in silico* benchmark.

Following the design of the protein’s backbone structure, an amino acid sequence that folds into the desired structure needs to be identified. This inverse folding problem has been addressed by ProteinMPNN,^46^ a graph neural network that outperforms previous tools such as Rosetta in terms of accuracy. Expanding on this framework, LigandMPNN^47^ enables the design of sequences that bind specific ligands. This is highly interesting for enzyme design as it can facilitate the design of active sites or cofactor binding regions. In this regard, it is also noteworthy that recent structure-prediction models like AlphaFold3,^48^ Chai-1,^49^ or Boltz-1 ^50^ possess the capability to predict the structure of protein–ligand complexes. These tools promise to further enhance the design process and provide an alternative to classical protein–ligand docking methods.

In parallel to these structure-focused protein design strategies, a number of ML models that generate new protein sequences without explicitly considering structure have emerged in recent years. Among these, transformer-based protein language models (pLMs) trained on large data sets of natural protein sequences have demonstrated the ability to generate new proteins that are only distantly related to the ones in the training data sets.^51^ A challenge lies in steering these unsupervised models toward generating proteins with desired properties, such as specific enzymatic activities. A notable step in this direction is ZymCTRL,^52^ which has been trained on enzyme sequences as well as corresponding EC numbers. Moreover, ESM3 ^53^ is a multimodal pLM that integrates information from protein sequences, structures, and functional annotations. It enables numerous tasks, including the scaffolding of active sites or function prediction from sequence alone. In addition, it has been demonstrated that pLMs can be fine-tuned to produce desired outputs by means of reinforcement learning,^54,55^ for example using supervised models trained on experimental activity data. Despite their ability to generate new sequence variants for native enzymatic activities, such as highly diverse lysozymes,^51^ the use of pLMs for the design of entirely new enzymes (e.g., catalyzing non-natural reactions) remains to be demonstrated.

The development of ML-based design routines has led to noteworthy advances over previous enzyme design efforts. Most notably, recent methodologies enable the placement of catalytic residues within *de novo* backbones with very high accuracy, thus addressing one of the primary limitations of initial Rosetta-based design routines. However, to obtain highly active enzymes, it is critical to start the design process from a catalytically competent theozyme. In a recent study, the catalytic tetrad of an experimentally optimized retro-aldolase was placed in *de novo* backbones with custom substrate pockets using a diffusion-based pipeline.^56^ The resulting designs exhibited remarkable accuracy and displayed activities that exceeded those of earlier computationally designed retro-aldolases by several orders of magnitude. However, accomplishing similar outcomes for novel reactions will require more advanced pipelines for computing highly accurate theozymes.

While transition-state stabilization is a basic tenet of enzymatic catalysis, the findings from the field of enzyme design underscore previous observations that this factor alone is not sufficient to rationalize the formidable activity of enzymes. Another critical aspect is the influence of conformational dynamics and active-site preorganization.^57^ While design routines typically operate on static structures, it is more accurate to consider the ensemble of structures that an enzyme can adopt in solution. Some structures in this ensemble will be more catalytically competent than others, and mutations that improve preorganization by shifting the distribution toward the competent conformation can have a substantial impact on activity.^58^ Predicting which mutations will trigger the desired conformational change is challenging, although some strategies based on MD simulations in combination with the correlation-based method Shortest Path Map (SPM) have been developed.^59,60^ Preorganization is frequently assessed by running nanosecond-time scale MD simulations. Due to the high computational cost of MD simulations, there is growing interest in approximating such simulations using ML tools. For example, AlphaFold2 has been adapted to predict multiple conformations of a protein,^61^ and the deep learning model BioEmu^62^ has been developed to emulate protein equilibrium ensembles on consumer-grade hardware.

Many enzymes undergo multiple conformational changes during the course of a catalytic cycle, which poses a substantial challenge for enzyme design. A notable step toward multistate enzyme design has recently been reported for serine hydrolases that rely on a four-step reaction mechanism.^63^ In this instance, researchers placed a catalytic triad and oxyanion hole into *de novo* backbones using RFdiffusion and subsequently filtered designs using PLACER (Protein–Ligand Atomistic Conformational Ensemble Resolver), a neural network that predicts conformational ensembles of proteins and small molecules. Assessing the preorganization in each step of the catalytic cycle using PLACER increased the design success rate substantially and resulted in enzymes with noteworthy catalytic efficiencies of up to 2.2 × 10^5^ M^–1^ s^–1^.

Another important facet of enzymatic catalysis is the influence of electrostatic effects. Over the past decades, it has become increasingly clear that enzymes can produce strong electric fields that promote the reaction.^64–66^ As electric fields over individual bonds can be computed quickly, there is potential to further enhance design workflows by considering such fields. This notion is supported by the successful *in silico* optimization of a designed Kemp eliminase based on electric fields.^67^

The examples provided above illustrate that enzyme design has made remarkable progress in terms of design accuracy. Nevertheless, a major bottleneck persists in the challenge of predicting the most active variants among designed enzymes. Addressing this challenge will require a computational analysis of factors such as conformational dynamics and electric fields, which can be used to filter designs and thus further reduce the number of variants that need to be tested to identify active enzymes. Even better results may be obtained by implementing design and filtering in an iterative manner, such that final designs are optimized with regard to catalytically relevant properties.^68^

### Enzyme Engineering

The past decades have witnessed major progress in enzyme engineering, largely enabled by directed evolution and the development of high-throughput screening platforms. However, the vastness of protein sequence space, combined with the propensity to become trapped in local optima, renders traditional approaches to exploring fitness landscapes inefficient and prone to suboptimal outcomes. To address these challenges, numerous computational tools have been developed to propose mutations or identify hot spots for improving properties such as activity, thermostability, and solubility. These tools frequently leverage evolutionary information or biophysical calculations and increasingly take advantage of ML algorithms. For example, MutCompute^69^ is a convolutional neural network that can identify amino acids that are not optimized for their local chemical microenvironment. Mutations suggested by this tool improved the activity of a PET-degrading enzyme at ambient temperatures while keeping the experimental effort modest.^70^ This kind of zero-shot enzyme engineering—i.e., approaches that do not rely on functional experimental data—shows great promise, but is likely most powerful in combination with directed evolution or supervised ML strategies.^71^

While screenings over multiple rounds are typically still required, ML can help to make these more efficient. The predictive power of sequence-function data sets has long been recognized,^72,73^ enabling the prediction of properties for untested variants and thereby guiding engineering campaigns. ML models with their ability to capture complex, nonlinear relationships are well suited to this task. Consequently, machine learning-assisted directed evolution (MLDE) has gained substantial attention in recent years.^74–80^

MLDE treats enzyme engineering as a supervised learning problem: starting from an initial sequence-function data set, a model is trained to predict function (often activity) from the amino acid sequence. Subsequently, this model can be used to design a new library of promising variants for experimental testing. This process can be iterated to improve the model and explore the sequence space in a model-guided fashion (referred to as active learning).^12,81^ Using deep-mutational scanning data sets, it has been demonstrated that MLDE can identify desired variants faster and more reliably than classical directed evolution strategies.^82^ However, in practice the success of MLDE campaigns depends on numerous factors, from the choice of suitable encodings and model architectures to the data set size and quality. Countless encodings of amino acid sequences as well as model architectures have been suggested in the literature, and while a few comparative studies have been performed,^83,84^ the best choice is very much case-dependent. Similarly, the required size of the training data set varies, but a few dozen to a few hundred data points have frequently been reported to be sufficient to develop useful models.^74–78^ However, larger data sets are likely to result in more reliable models. It is also important to note that some data sets are more informative as training data than others.^84^ Strategies aiming to increase the information density include free-energy calculations to exclude destabilizing mutations,^85^ as well as advanced zero-shot predictors based on pLMs.^86^ Noise and measurement errors can likewise impact model performance substantially.^80^ Once a model has been developed, a sampling strategy that balances exploration and exploitation needs to be selected for subsequent rounds. Frequently, active-learning algorithms relying on Bayesian Optimization are applied to achieve this balance.^74,87^

While current tools are often sufficient to perform successful MLDE campaigns, further advances with respect to informative encodings and zero-shot prediction are desirable, particularly for settings where little training data are available. Moreover, most models exhibit limitations in their capability to reliably extrapolate beyond the training distribution or to generalize to related reactions. Therefore, transfer learning strategies that leverage evolutionary information,^88^ predicted biophysical properties,^89^ or performance data on related, yet easily screenable reactions are interesting areas of research.

Most studies to date target a rather small search space and thus do not deliver on the promise of thorough protein engineering beyond the capabilities of advanced high-throughput screening methods. Similarly, multiobjective optimization remains underexplored, despite the need for enzymes to meet a range of requirements beyond the target activity for industrial application. Thus, ample opportunities remain to further augment enzyme engineering with the help of ML.

### Experimental Technologies

Besides breakthroughs in computer science and increased computing power, the progress in ML-driven enzyme development has also been enabled by advances on the experimental side. To fully harness the potential of ML-driven workflows, further progress in experimental methods and approaches is imperative.

One crucial component is the remarkable decline in the cost of DNA synthesis and sequencing, as it provides both training data and experimental validation on a scale that was previously out of reach. Nonetheless, the costs associated with synthesis and sequencing can still constrain library sizes and impede the widespread adoption of ML-based methodologies. For example, while ML can reduce the screening effort during enzyme engineering, the additional sequencing costs may render traditional strategies, where sequence information is only obtained for selected hits, more economically viable. A potential solution is to rely on next-generation sequencing (NGS), which is highly cost-effective on a per-variant basis, but may require bespoke barcoding strategies.^80,90^ With regard to library generation, strategies to assemble full-length genes from oligonucleotide pools are a way to reduce costs.^91,92^ Moreover, variational synthesis is a cost-effective way to create extremely large libraries that are the output of a generative model with knowledge of chemical DNA synthesis.^93^

It is important that requirements and expectations are discussed early on in a collaboration, which may involve planning preliminary experiments.

Testing large numbers of enzyme variants can be labor-intensive and time-consuming. Accordingly, screening methods that reduce manual labor and allow for highly parallelized experimentation are desirable for data-driven enzyme development. A possible solution is to make use of lab automation, which also holds the promise of enhanced reproducibility.^94^ However, setting up an automated screening procedure can be a time-consuming endeavor in itself. More flexible and modular lab automation solutions could greatly facilitate the adoption of automation and the generation of data for MLDE and other purposes. Moreover, integrating automation with computational pipelines or AI agents may eventually enable the development of enzymes with no or minimal human intervention.^95^ In fact, the repetitive nature of enzyme screenings may make the implementation of such “self-driving” laboratories easier than in other fields of research.

Beyond automation, several methods have been developed to screen and characterize enzyme variants at scale, yielding data sets that are highly attractive for ML applications. For example, enzymatic reactions can be miniaturized and parallelized on microfluidic chips,^96^ and NGS can be combined with cell sorting or DNA recorders to perform high-throughput sequence-function mapping.^97^ However, adapting such methods to specific enzymatic reactions is often challenging. Consequently, the development of a high-throughput sequence-function mapping strategy that is broadly applicable to enzymes remains an outstanding challenge. Biosensors linked to cell growth or DNA modification as a readout could yield very large data sets in a relatively straightforward manner, but as suitable sensors are lacking for most relevant products, better biosensor design methods are required as a first step. Droplet microfluidics coupled with mass spectrometry could be another potent and flexible method; however, maintaining the genotype-phenotype linkage is crucial.^98^ It should also be considered that data from high-throughput methods is frequently more noisy than results from classical well-plate assays, which may pose a challenge for the development of accurate ML models.^99^

## Challenges in Adopting and Advancing ML-Based Workflows

Besides the technical challenges outlined above, the implementation and development of ML-based tools and workflows can be hindered by numerous practical challenges, for example related to the accessibility of published tools or the lack of a common language between wet lab and dry lab teams. While seemingly mundane, such issues can have a profound impact on the success of projects and the speed of progress in the field.

Currently, experimentalists aiming to integrate ML into their workflows can face a number of hurdles. While some tools are available via special web servers or Colab implementations, others are only available on GitHub and may be challenging to use without computational expertise. Experience is also required with regard to data processing, parameter choices, understanding mathematical models, and the interpretation of results. Efforts from publicly funded organizations to build and maintain accessible implementations of popular tools and provide the necessary training are needed to remove such barriers.

Integrating enzyme design and engineering into a unified pipeline could merge bold leaps in sequence space with fine-grained optimization.

In many cases, progress in the field also depends on the effective collaboration between experimentalists and computer scientists. As most experimentalists and computer scientists possess limited training in ML and biochemistry, respectively, such collaborations can be prone to misunderstandings and unrealistic expectations on both sides. For example, the ideal data set for ML may differ substantially from what can be readily generated in the lab, and proper handling of the data requires an understanding of how the data has been generated. Moreover, it is not uncommon that experimental conditions vary from one screening round to the next (for example due to unreliable equipment or the desire to continuously optimize conditions), but such variations pose a challenge for the computational analysis. For these reasons, it is important that such requirements and expectations are discussed early on in a collaboration (see Figure 2 for a “collaboration checklist”). This may also involve planning preliminary experiments to determine whether the data that can be generated is suitable for computational analysis. Despite such challenges, collaborations between wet lab and dry lab teams are often fruitful and will continue to play a crucial role in biocatalysis. Consequently, it will be important to update curricula at universities to reflect the growing importance of ML in the life sciences and provide future generations of scientists with the required interdisciplinary training.

**Figure 2:**
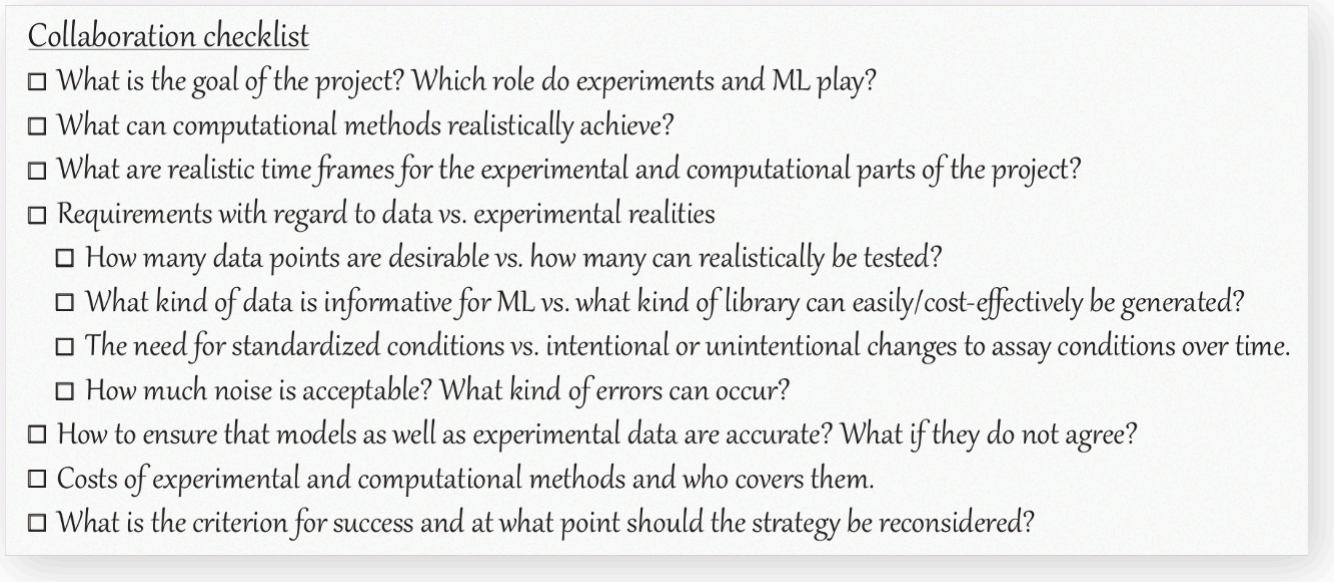
Checklist for collaborations between wet lab and dry lab teams.

## Future Directions

The computational and experimental toolbox available to enzyme designers and engineers has grown at a rapid pace in recent years, and ML now plays a pivotal role in enzyme development pipelines. While some ML tools and strategies (e.g., for protein structure prediction) can be considered mature and well-established, others require further development and validation.

Democratizing access to ML-driven enzyme development will require open-source tools, centralized biofoundries, and low-cost automation.

Frequently, the development of enzyme-specific ML models is hindered by the limited availability of large, high-quality data sets on enzymatic properties across diverse enzyme families, resulting in tools with limited scope and generalizability. Initiatives from funding agencies to support the generation and sharing of such data sets could therefore be highly valuable. Ideally, such data sets should provide information on multiple enzymatic properties (e.g., catalytic parameters, expression yield, thermostability) across various substrates and reactions conditions. In addition, standardized protocols and comprehensive metadata reporting are critical for ML applications. Models trained on such data could enable more accurate zero-shot predictions, which would be useful in a wide range of scenarios. For example, even a coarse-grained activity estimate can greatly reduce the number of variants that need to be tested during enzyme discovery or following *de novo* design. Data sets on multiple properties could also accelerate the development of multimodal models, which promise to be powerful tools, for example with respect to multiobjective optimization.

Further advancements could come from pipelines that integrate various tools and data sources into combined workflows. In particular, tightly integrating enzyme design and engineering into one unified pipeline can merge the capability of generative models to perform large jumps in sequence space with the local optimization provided by engineering methods. Moreover, such approaches enable iterative improvements to design models based on experimental feedback.

As the number of computational tools continues to grow, it will be crucial to rigorously test and validate them to build trust within the community. In the case of protein structure prediction, the Critical Assessment of Structure Prediction (CASP) competition provided an independent evaluation, quickly fostering confidence in the structure prediction tools. In the case of protein design or engineering, systematic tests by means of comparative studies or competitions are less established. Just recently, a number of competitions have emerged^100^ and benchmarks (such as ProteinGym^101^) have been put forward. Such efforts could be highly valuable by providing guidance as to which tools and strategies are the most powerful and reliable within this rapidly evolving field. Moreover, it will be important to address biases in training data sets and remove data leakage between training and test sets^102^ to provide a reliable performance evaluation.

Progress on the experimental side will also continue to be important to the field, for example with respect to the cost, throughput, and reproducibility of data acquisition. In addition, active learning strategies would benefit from shorter feedback loops between computation and experiment, which could be facilitated by advances in DNA synthesis (such as enzymatic oligo synthesis and DNA assembly^103^) and lab automation platforms (such as self-driving labs or low-cost benchtop systems). In addition, cell-free gene synthesis and expression systems, possibly in combination with microfluidics, can markedly accelerate the build and test phase of design-build-test-learn cycles.^96,104,105^ However, it should be considered that differences in expression and reaction conditions between screening and the final application are often problematic. Models that account for such differences and predict performance across a range of conditions could mitigate such problems and facilitate the industrial scale-up process. In particular, multimodal models that integrate sequence and structure with stability and solubility data hold promise for capturing these complexities more comprehensively. In the long term, advanced biophysical simulations (or ML-based approximations thereof) could substantially reduce the need for experimental testing, but such approaches are still in their infancy in the context of biological systems.

Numerous computational and experimental methods are available for the different phases of the DBTL cycle. When selecting which methods to use, a challenge lies in aligning the capabilities and requirements of these methods with each other and with external constraints, such as project budgets and timelines. Moreover, the great diversity of enzymes and enzymatic reactions make the development of universal platforms challenging, both from the computational and experimental perspective. Nonetheless, it is possible to establish broadly applicable pipelines that integrate the discovery or design of candidate sequences with DNA synthesis, expression, and activity assays as well as iterative refinements in a largely or fully automated manner. Depending on the available throughput, different strategies can be envisioned (Figure 3). If only a small number of variants can be experimentally tested, virtual screenings (e.g., using docking methods or MD) can be performed to identify the most promising variants, and transfer learning and foundation models can be leveraged to obtain better predictions. If a higher throughput is possible, cost-efficient DNA synthesis methods become important, and the resulting data can be used to train large, possibly multimodal models.

**Figure 3:**
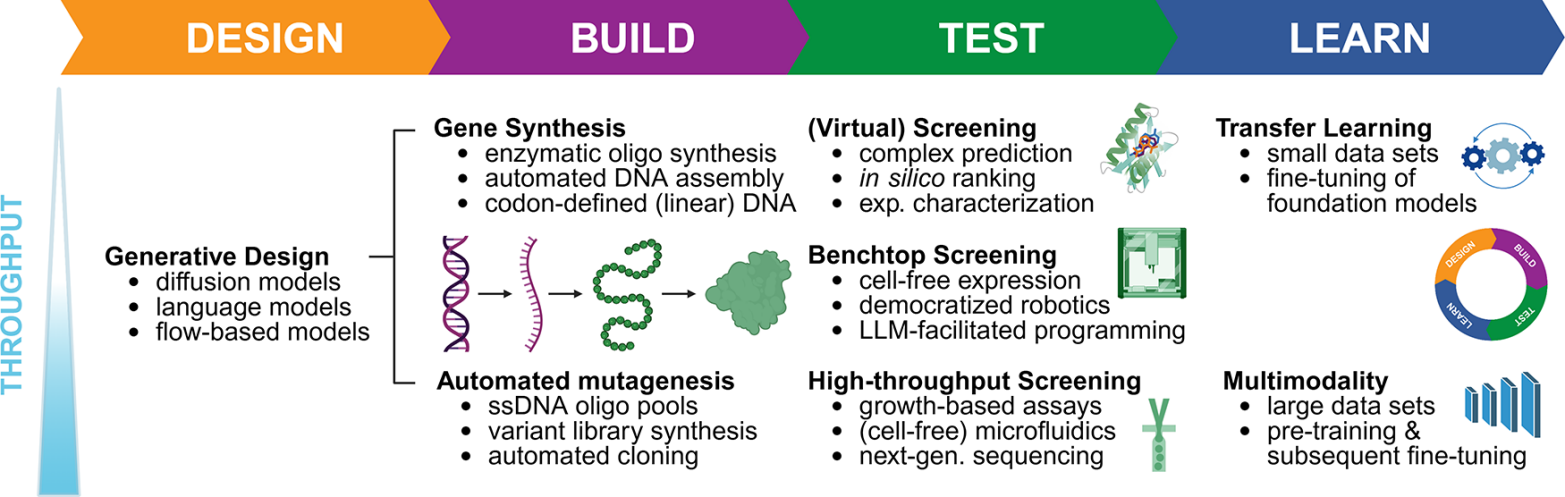
Enabling technologies and synergistic strategies for every stage of the design-build-test-learn cycle.^8^ As a strategy for designing novel enzyme variants, generative design (relying on fine-tuned diffusion, language, or flow-based models) is proposed for both low- and high-throughput scenarios. Depending on the available experimental throughput, genes encoding the predicted variants are either directly synthesized (for example, via enzymatic oligo synthesis and assembly) or generated through automated mutagenesis and cloning strategies (for example, relying on ssDNA oligo pools). In low-throughput scenarios, virtual prescreening (for instance, using AI-based protein–ligand complex predictions and MD simulations) offers substantial potential to narrow down the number of variants to be tested experimentally. Low-cost benchtop robotics platforms and cell-free expression systems are viewed as enabling technologies for democratized medium-throughput screening, while further advances in growth-based assays and microfluidics would qualify these technologies for future high-throughput screening applications. This would be particularly valuable for generating large data sets suitable for pretraining multimodal models. Low-throughput scenarios can benefit from pretrained foundation models, as fine-tuning them on specialized data sets enables transfer learning to novel downstream tasks even when data set sizes are limited.

As setting up such “lab-in-a-loop” operations requires substantial financial resources and expertise across multiple disciplines, a critical question will be how to democratize access to state-of-the-art technologies. Centralized biofoundries could play an important role in this regard, in particular if access can be provided via simple web interfaces. Low-cost automation platforms with accessible, chatbot-assisted programming interfaces offer a complementary, decentralized solution by facilitating the adoption of medium-throughput benchtop automation platforms. The latter would benefit from streamlined (cell-free) gene synthesis and expression (*vide supra*) to reduce the complexity of liquid handling and experimental procedures. In parallel, the development and dissemination of open-source computational tools will be essential to ensure that powerful ML models are broadly accessible, reducing dependence on proprietary platforms and enabling transparent, community-driven innovation.

As both computational and experimental technologies become more mature and powerful, it will be time to move from low-hanging fruit to more challenging but potentially impactful targets. Biocatalysis has long been hailed as an important puzzle piece in the urgently required transition toward a greener economy, and a concerted effort to develop enzymes for impactful “dream reactions” (e.g., new CO_2_ fixation routes or upcycling of waste products) could be instrumental in realizing this potential.
